# Surgeon seniority and performance in breast-conserving surgery for non-palpable lesions: evidence from a multicenter study

**DOI:** 10.1016/j.breast.2026.104736

**Published:** 2026-02-09

**Authors:** Fabio Corsi, Maria Luisa Gasparri, Sara Albasini, Matilde Pelizzola, Carlo Morasso, Giulia Armatura, Alessandro Asaro, Virginia Casati, Corrado Chiappa, Virginia Coli, Francesca Combi, Andrea Cuccaro, Angelica Della Valle, Raimondo Di Giacomo, Secondo Folli, Massimo Maria Grassi, Stefano Mancini, Ilaria Maugeri, Andrea Papadia, Laura Roveda, Francesca Rovera, Silvia Segattini, Adele Sgarella, Claudio Siani, Norma Stefenelli, Francesco Valenti, Simone Zanotti

**Affiliations:** a Department of Biomedical and Clinical Sciences, University of Milan, Milan, Italy; b Istituti Clinici Scientifici Maugeri IRCCS, Pavia, Italy; c Dep. of Gynecology and Obstetrics, Ente Ospedaliero Cantonale (EOC), Ospedale Regionale di Lugano, Lugano, Switzerland; d Faculty of Biomedicine, University of Italian Switzerland (USI), Lugano, Switzerland; e Centro di Senologia della Svizzera Italiana (CSSI), Ospedale Italiano di Lugano, Lugano, Switzerland; f General Surgery Residency Program, University of Milan, Milan, Italy; g Chirurgia Plastica e Senologica, Azienda Provinciale per i Servizi Sanitari di Trento, Italy; h Breast Surgery Unit, Department of Surgery, ASST Fatebenefratelli - Sacco, Luigi Sacco University Hospital, Milano, Italy; i SC Breast Unit, ASST-Settelaghi di Varese, Varese, Italy; j UOSD Oncologia Chirurgica Ricostruttiva della Mammella, PO "F.Lotti" - USL Toscana Nordovest – Pontedera, Italy; k Division of Breast Surgical Oncology, Department of Medical and Surgical, Maternal-Infantile and Adult Sciences, University Hospital of Modena, Modena, Italy; l Breast Unit, IRCCS Azienda Ospedaliero-Universitaria, Bologna, Italy; m General Surgery 3‐ Breast Surgery, Department of Surgery, Fondazione IRCCS Policlinico San Matteo, Pavia, Italy; n I.R.C.C.S. “G. Pascale”, Napoli, Italy; o Breast Unit, Surgery, Fondazione IRCCS Istituto Nazionale dei Tumori, Milan, Italy; p Breast Unit, Humanitas Gavazzeni e Castelli, Bergamo, Italy

**Keywords:** Breast-conserving surgery, Non-palpable breast lesions, Surgeon seniority

## Abstract

Breast-conserving surgery (BCS) for non-palpable lesions is technically demanding, often performed by surgical trainees under supervision. Despite extensive literature evaluating localization techniques, only few studies have directly examined the role of surgeon seniority in determining surgical performance in this specific setting.

We conducted a retrospective multicenter analysis (LOCALIZATION01 study, NCT05942105) including 3,195 patients who underwent BCS for non-palpable breast lesions between 2016 and 2024 across 12 Breast Units. Four localization techniques were used: wire-guided (WGL), radioguided occult lesion localization (ROLL), magnetic seed localization (MSL), carbon localization (CL). Outcomes included margin status, calculated resection ratio (CRR), operative time, and complications, stratified by surgeon seniority (attending vs resident).

Most procedures were performed by attending surgeons (89.3%, n = 2,855) compared to residents (10.7%, n = 340). Margin positivity rates didn't differ significantly across localization techniques (e.g., ROLL 3.7% vs 2.3%, p = 0.30; MSL 9.4% vs 4.4%, p = 0.11, WGL 2.7% vs 5.9 p = 0.16, CL 0% vs 9.6% p = 0.10). Residents achieved better CRR in ROLL procedures (2.0, IQR 3, [1-47] vs 2.8, IQR 5, [1-78], p = 0.006), but had longer operative times, particularly with SLNB (e.g., MSL 60 min, IQR 31, [37-98] vs 55 min, IQR 20, [18−180], p = 0.02). Complication rates were low and comparable between groups.

In a supervised setting, surgeon seniority doesn't significantly impact margin status, complication rates, or overall oncologic safety in BCS for non-palpable breast lesions. Localization methods with high reproducibility, such as ROLL and MSL, may mitigate the influence of surgical inexperience. These findings support the safe involvement of trainees in BCS under adequate supervision.

## Highlights

• Non-palpable breast lesions requires accurate localization methods.
• Surgeon seniority did not significantly affect margins, complications, or safety.
• Residents achieved better CRR with ROLL but had longer operative times overall.
• ROLL and MSL may offset inexperience, supporting safe trainee involvement.

## 1 Introduction

Breast-conserving surgery (BCS) for non-palpable breast lesions represents a complex surgical task, requiring accurate lesion localization, precise excision, and preservation of healthy tissue to ensure both oncologic safety and satisfactory cosmetic outcomes [1,2]. In academic and teaching hospitals, many BCS procedures are performed by surgical residents under the supervision of attending surgeons. While progressive autonomy is a cornerstone of surgical training, concerns persist regarding the potential impact of operator experience on key outcomes, including surgical margin status, complication rates, and the need for reintervention.

The actual influence of trainee involvement on surgical quality remains insufficiently defined [3]. Achieving negative margins is critical not only for oncologic control but also to reduce healthcare costs, limit the volume of resected tissue, and avoid the need for additional surgeries or conversion to mastectomy [[4], [5], [6]].

Over the years, several localization strategies have been developed to facilitate the excision of non-palpable breast lesions [7,8]. Wire-guided localization (WGL) has long been considered the standard technique, although it carries limitations related to patient discomfort, same-day scheduling, and risk of wire displacement [1,[9], [10], [11]]. Radioguided occult lesion localization (ROLL) has emerged as a valid alternative [12], with some studies reporting higher rates of negative margins and shorter operative times [13,14]. More recently, wireless methods such as magnetic seed localization (MSL) have gained popularity for their logistical advantages and oncologic safety [[15], [16], [17], [18]], while carbon localization (CL) remains a simple, cost-effective option in selected centres which involves the injection of a sterile activated carbon suspension that leaves a visible track lasting several days [19,20]. These approaches underscore how localization choice may influence both surgical precision and learning opportunities for trainees.

Nevertheless, regardless of the technique employed, surgical margin status remains the ultimate determinant of oncologic adequacy [[21], [22], [23]]. Positive margins are consistently associated with increased local recurrence risk and higher rates of re-excision, affecting up to 14% of patients even in contemporary series [21,24]. Given these implications, understanding whether surgeon experience independently contributes to margin status—beyond the localization method—is crucial for both patient safety and surgical education.

Despite an extensive literature search evaluating localization techniques, only few studies have directly examined the role of surgeon seniority in determining surgical performance in this specific setting [3,23,25,26]. To address this gap, we conducted a large multicenter retrospective analysis of 3,195 patients undergoing BCS for non-palpable lesions using different localization methods. First, we conducted a survey across several Breast Units accredited within the National Healthcare System to identify the most frequently used preoperative localization techniques for non-palpable breast lesions. We then compared the performance of attending surgeons with that of surgical trainees, with specific reference to surgical margin status, resection volumes relative to lesion size, operative times, and postoperative complications.

## 2 Materials and Methods

### 2.1 Study type and design

A retrospective analysis was performed within the multicenter LOCALIZATION01 study (Institute's approval: CE2776, ClinicalTrial.gov ID: NCT05942105), including all patients who underwent breast-conserving surgery for non-palpable breast lesions at 11 major Breast Units in Italy and one in Switzerland. Data were collected in accordance with a study protocol approved by the Ethical Committee of the coordinating institution (Istituti Clinici Scientifici Maugeri, Pavia, Italy) and of the participating centres. All patients underwent BCS, with or without concomitant axillary procedures, and non-palpable breast lesions were localized using the standard techniques adopted at each participating center. Clinical data and medical history were recorded for every case. Margin status of the excised specimens was assessed according to widely accepted criteria: ‘no ink on tumor’ for invasive carcinoma and a 2-mm clearance for carcinoma in situ [27]. To evaluate the extent of unnecessary tissue removal, the Calculated Resection Ratio (CRR) was employed [28] (defined as the ratio between the actual resection volume — calculated as an ellipsoid using the three specimen dimensions — and the optimal resection volume — estimated as the spherical volume of the lesion plus an additional 1 cm margin). For each procedure, the level of the operating surgeon (attending or resident) was recorded. For attending surgeons, we considered all operative reports in which they were identified as the primary operators. First-year residents training program were excluded, as their limited level of experience may have influenced the outcomes of the parameters under analysis. The primary outcome was the rate of negative surgical margins in BCS performed by attending versus resident surgeons across different localization techniques. Secondary endpoints included comparisons of CRR, surgical time and postoperative complications between the two groups.

### 2.2 Patients’ population

A total of 3,195 patients who underwent breast-conserving surgery for non-palpable breast lesions between 2016 and 2024 across 12 Breast Units were included. Lesions diagnosed by core biopsy as B3/C3, B4/C4, B5/C5 were eligible. Patients who had received neoadjuvant chemotherapy were excluded.

Patients were categorized according to the localization technique used by each center. The techniques detected in the LOCALIZATION01 study were: wire-guided localization (WGL), radioguided occult lesion localization (ROLL), magnetic seed localization (MSL), and carbon localization (CL).

### 2.3 Statistical analysis

Variables were reported as medians with interquartile ranges (IQR) and ranges, or as absolute frequencies and percentages. Comparisons of categorical variables were performed using the χ^2^ test or Fisher's exact test, as appropriate, while continuous variables were analysed with the Pairwise Two-Sided Multiple Comparison test (Dwass–Steel–Critchlow–Fligner method). Statistical significance was set at p < 0.05 (two-tailed). All analyses were conducted using SAS software, version 9.4 (SAS Institute Inc., Cary, USA).

## 3 Results

### 3.1 Characteristics of the study cohort

In the analysed cohort of 3,195 patients, as shown in Table 1 and relative Table 2 for comparison, the median patient age ranged from 57 years old (IQR 19, [21-91]) in the ROLL group to 63 years (IQR 17, [30-91]) in the WGL group, with intermediate values of 60 years (IQR18, [20-88]) for MSL and 61 years (IQR 18, [35-82]) for CL. Across all localization techniques, the majority of patients were postmenopausal (ROLL 73.3%, MSL 77.2%, WGL 83.6% and CL 73.8%) and had a BMI <30 (ROLL 90.5%, MSL 83.9%, WGL 86%, CL 74.4%). With respect to lesion morphology, nodules (ROLL 47.3%, MSL 62.2%, WGL 66.4%, CL 53.8%) represented the most frequent presentation, followed by microcalcifications (ROLL 45.6%, MSL 28.8%, WGL 25.6%, CL 35.3%) and, less commonly, distortion (ROLL 7.1%, MSL 9%, WGL 8%, CL 10.9%). Most lesions were classified as B5 at core biopsy (ROLL 70.8%, MSL 83%, WGL 96.3%, CL 90%).

**Table 1:** Baseline characteristics of the entire cohort.

Characteristics	ROLL n = 985	MSL n = 551	WGL n = 1078	CL n = 581
**Age at diagnosis (years)**∗	57 (19) [21-91]	60 (18) [20-88]	63 (17) [30-91]	61 (18) [35-82]
**Hormonal status**				
Fertile	227 (26.7)	109 (22.8)	177 (16.4)	152 (26.2)
Menopause	623 (73.3)	369 (77.2)	901 (83.6)	428 (73.8)
**Patients' Body Mass Index (BMI)**				
<30	789 (90.5)	250 (83.9)	927 (86)	348 (74.4)
≥30	83 (9.5)	48 (16.1)	151 (14)	120 (25.6)
**Lesion morphology**				
Nodule	459 (47.3)	317 (62.2)	716 (66.4)	311 (53.8)
Microcalcification	442 (45.6)	147 (28.8)	276 (25.6)	204 (35.3)
Distortion	69 (7.1)	46 (9)	86 (8)	63 (10.9)
**Lesion size on imaging (mm)**∗	10 (8) [1-120]	9 (5) [1-50]	10 (7) [1-120]	10 (8) [2-80]
**Cyto/histological result (CB)**				
B3/C3	273 (27.9)	84 (15.4)	24 (2.2)	46 (7.9)
B4/C4	13 (1.3)	9 (1.6)	16 (1.5)	12 (2.1)
B5a/B5b/C5	692 (70.8)	453 (83)	1038 (96.3)	523 (90)
**Post-procedure hematoma**				
No	777 (91.7)	470 (96.9)	1056 (98)	532 (95.9)
Yes	70 (8.3)	15 (3.1)	22 (2)	23 (4.1)
**Surgeon who performed resection**				
Attending surgeon	824 (83.6)	497 (90.2)	854 (85.2%)	552 (95%)
Resident	161 (16.4)	54 (9.8)	148 (14.8%)	29 (5%)
**Surgical radicalization**				
No	504 (51.2)	135 (24.5)	460 (42.7)	67 (11.5)
Yes	481 (48.8)	416 (75.5)	618 (57.3)	514 (88.5)
**Type of axillary surgery**				
None	461 (46.9)	169 (30.8)	196 (18.2)	174 (30)
SLNB only	473 (48.1)	353 (64.3)	815 (75.6)	382 (65.7)
ALND	50 (5.1)	27 (4.9)	67 (6.2)	25 (4.3)
**pT**				
No (benign lesion)	224 (23.7)	69 (13.5)	6 (0.6)	26 (4.6)
pT1	465 (49.2)	346 (67.6)	773 (72)	353 (62.2)
pT1mic	26 (2.8)	7 (1.3)	17 (1.6)	11 (1.9)
pT2	35 (3.7)	5 (1)	59 (5.5)	20 (3.5)
pT3	0 (0)	0 (0)	1 (0.1)	0 (0)
pT4	0 (0)	0 (0)	0 (0)	1 (0.2)
pTIS	195 (20.6)	85 (16.6)	217 (20.2)	157 (27.6)
**Surgical margins**				
Not involved	956 (97.4)	522 (95.1)	1018 (94.5)	526 (90.8)
Involved	25 (2.6)	27 (4.9)	58 (5.4)	53 (9.2)
**CRR**∗	2.7 (4.9) [1-77.7]	2.7 (3.6) [1-90.7]	2.4 (3.3) [1-43.7]	3 (3.6) [1-50]
**Surgery time (min)**∗				
BCS only∗	50 (25) [14−200]	48 (26) [10−208]	70 (32) [35−180]	55 (29) [20−250]
BCS with SLNB∗	60 (22) [25−189]	55 (20) [18−180]	95 (36) [40−225]	75 (40) [19−180]
BCS with ALND∗	100 (45) [50−212]	75 (55) [30−165]	143 (80) [95−210]	95 (60) [30−180]
**Surgical complications**				
No	907 (95.9)	522 (95.6)	1053 (97.9)	483 (83.4)
Yes	39 (4.1)	24 (4.4)	23 (2.1)	96 (16.6)

Values are n (%) unless otherwise indicated; values.

∗ are median (i.q.r.) [range]. ROLL = Radioguided Occult Lesion Localization; MSL = Magnetic Seed; CB = core biopsy; WGL= Wire Gide Localization; CL= Carbon Localization; BCS= Breast-conserving surgery; SLNB = sentinel lymph-node biopsy; ALND = axillary lymph-nodes dissection.

**Table 2:** Comparisons (p-values) for each method and variable related to Table 1.

	Age			Hormonal status			BMI			Lesion morphology		
	MSL	WGL	CL	MSL	WGL	CL	MSL	WGL	CL	MSL	WGL	CL
**ROLL**	0.003	<0.0001	0.003	0.12	<0.0001	0.83	0.002	0.002	<0.0001	<0.0001	<0.0001	0.0001
**MSL**		0.02	1.00		0.003	0.20		0.36	0.002		0.25	0.02
**WGL**			0.01			<0.0001			<0.0001			<0.0001

	**Lesion size on imaging**			**Cyto/histological result (CB)**			**Post-procedure hematoma**			**Surgeon who performed resection**		
	**MSL**	**WGL**	**CL**	**MSL**	**WGL**	**CL**	**MSL**	**WGL**	**CL**	**MSL**	**WGL**	**CL**
**ROLL**	0.14	<0.0001	0.002	<0.0001	<0.0001	<0.0001	0.0002	<0.0001	0.002	0.0004	0.33	<0.0001
**MSL**		<0.0001	<0.0001		<0.0001	0.0004		0.21	0.37		0.005	0.002
**WGL**			1.00			<0.0001			0.01			<0.0001

	**Surgical radicalization**			**Type of axillary surgery**			**pT**			**Surgical margins**		
	**WGL**	**WGL**	**CL**	**MSL**	**WGL**	**CL**	**MSL**	**WGL**	**CL**	**MSL**	**WGL**	**CL**
**ROLL**	<0.0001	0.0001	<0.0001	<0.0001	<0.0001	<0.0001	<0.0001	<0.0001	<0.0001	0.01	0.001	<0.0001
**MSL**		<0.0001	<0.0001		<0.0001	0.82		<0.0001	<0.0001		0.69	0.006
**WGL**			<0.0001			<0.0001			<0.0001			0.003

	**CRR**			**Surgery time (BCS only)**			**Surgery time (BCS with SLNB)**			**Surgery time (BCS with ALND)**		
	**MSL**	**WGL**	**CL**	**MSL**	**WGL**	**CL**	**MSL**	**WGL**	**CL**	**MSL**	**WGL**	**CL**
**ROLL**	0.86	0.01	0.41	0.13	<0.0001	0.002	<0.0001	<0.0001	<0.0001	0.05	0.02	1.00
**MSL**		0.27	0.12		<0.0001	<0.0001		<0.0001	<0.0001		0.002	0.23
**WGL**			<0.0001			0.0004			<0.0001			0.05

	**Surgical complications**											
	**MSL**	**WGL**	**CL**									
**ROLL**	0.80	0.01	<0.0001									
**MSL**		0.01	<0.0001									
**WGL**			<0.0001									

ROLL = Radioguided Occult Lesion Localization; MSL = Magnetic Seed; CB = core biopsy; WGL= Wire Gide Localization; CL= Carbon Localization; BCS= Breast-conserving surgery; SLNB = sentinel lymph-node biopsy; ALND = axillary lymph-nodes dissection.

With regard to margin involvement, CL showed the highest rate (9.2%), which was significantly greater compared with ROLL (2.6%), MSL (4.9%), and WGL (5.4%) (all p < 0.05). The CRR was also highest in median for CL (3.0, IQR 3.6, [1-50]) compared with ROLL (2.7, IQR 4.9, [1-77.7]), MSL (2.7, IQR 3.6, [1-90.7]), and WGL (2.4, IQR 3.3, [1-43.7]), but statistical significance was observed only for the comparison between CL and WGL (p < 0.0001). Postoperative complication rates were likewise higher in CL (16.6%) compared with ROLL (4.1%), MSL (4.4%), and WGL (2.1%) (all p < 0.0001).

In terms of median operative time, MSL was the fastest technique (48 min, IQR 26, [10−208]) for BCS-only procedures, reaching statistical significance compared with CL and WGL (both p < 0.0001). Also for BCS with SLNB, MSL was significantly faster (55 min, IQR 20, [18−180]) than the other techniques (all p < 0.0001), while for BCS with ALND, ROLL and MSL were significantly faster (respectively 100 min, IQR 45, [50−212] and 75 min, IQR 55, [30−165]) than WGL (respectively p = 0.02 and p = 0.002).

In our cohort the majority of breast-conserving procedures were performed by attending surgeons (89.3%, 2.855 cases) compared to surgical residents (10.7%, 340 cases).

ROLL was the most frequently performed localization technique among residents (16.4%), compared to MSL (9.8%), WGL (14.8%), and CL (5%). The difference was statistically significant when compared to all other techniques (all p < 0.05), except for WGL (p = 0.33). Carbon localization was the least performed by residents (5%), also reaching statistical significance when compared to the other methods (all p < 0.05).

### 3.2 Surgery's outcomes by localization technique and surgeon

As reported in Table 3, no statistically significant differences in surgical margin status were observed between residents and attending surgeons across all localization techniques. However, a trend emerged, with residents exhibiting a higher rate of positive margins in MSL (9.4%), followed by ROLL (3.7%). In Table 4, we therefore evaluated the rate of involved margins across the four localization techniques, both for attending surgeons and residents, in microcalcifications and in nodules and distortion found no statistically significant differences (Nodules and distortion: ROLL p = 1.00, MSL p = 0.43, WGL p = 0.07, CL p = 0.38; Microcalcifications: ROLL p = 0.13, MSL p = 0.09, WGL p = 1.00, CL p = 0.61).

**Table 3:** Comparison of negative surgical margins after surgery according to localization technique and operating surgeon.

	ROLL			MSL			WGL			CL		
	Attending surgeon	Resident	p	Attending surgeon	Resident	p	Attending surgeon	Resident	p	Attending surgeon	Resident	p
**Surgical margins not involved**	801 (97.7)	155 (96.3)	0.30	474 (95.6)	48 (90.6)	0.11	802 (94.1)	144 (97.3)	0.16	497 (90.4)	29 (100)	0.10
**Surgical margins involved**	19 (2.3)	6 (3.7)		22 (4.4)	5 (9.4)		50 (5.9)	4 (2.7)		53 (9.6)	0(0)	

Values are n (%). ROLL = Radioguided Occult Lesion Localization; MSL = Magnetic Seed; WGL= Wire Gide Localization; CL= Carbon Localization.

**Table 4:** Comparison of negative surgical margins after surgery according to localization technique, operating surgeon and lesion morphology.

	Nodules and distortion											
	ROLL			MSL			WGL			CL		
	Attending surgeon	Resident	p	Attending surgeon	Resident	p	Attending surgeon	Resident	p	Attending surgeon	Resident	p
**Surgical margins not involved**	423 (97,9)	91 (97,8)	1.00	304 (95,9)	41 (93,2)	0.43	603 (95)	109 (99,1)	0.07	326 (91,8)	18 (100)	0.38
**Surgical margins involved**	9 (2,1)	2 (2,2)		13 (4,1)	3 (6,8)		32 (5)	1 (0,9)		29 (8,2)	0 (0)	

	**Microcalcifications**											
	**ROLL**			**MSL**			**WGL**			**CL**		
	**Attending surgeon**	**Resident**	**p**	**Attending surgeon**	**Resident**	**p**	**Attending surgeon**	**Resident**	**p**	**Attending surgeon**	**Resident**	**p**
**Surgical margins not involved**	367 (97,4)	60 (93,8)	0.13	131 (93,6)	5 (71,4)	0.09	199 (91,7)	35 (92,1)	1.00	169 (87,6)	10 (100)	0.61
**Surgical margins involved**	10 (2,7)	4 (6,3)		9 (6,4)	2 (28,6)		18 (8,3)	3 (7,9)		24 (12,4)	0 (0)	

Values are n (%) unless otherwise indicated; values ∗ are median (i.q.r.) [range]. ROLL = Radioguided Occult Lesion Localization; MSL = Magnetic Seed; WGL= Wire Gide Localization; CL= Carbon Localization.

Regarding the secondary outcomes (Table 5), analysis of the CRR showed that residents achieved better results compared to attending surgeons in the ROLL group (median CRR: 2.0, IQR 3, [1-47] vs 2.8, IQR 5, [1-78], p = 0.006), while a similar but non-significant trend was seen in MSL (median CRR: 2.3, IQR 4, [[1], [2], [3], [4], [5], [6], [7], [8], [9], [10], [11], [12], [13], [14], [15], [16], [17], [18], [19], [20], [21], [22], [23], [24], [25]] vs 2.7, IQR 3, [1-91], p = 0.77). In terms of surgical time, residents generally required equal or longer operative duration across all procedures (BCS alone, BCS with SLNB, BCS with ALND). The median operative duration was significantly shorter for attending surgeons in the BCS with SLNB subgroup for both MSL (55 min, IQR 20, [18−180], vs 60 min, IQR 31, [37-98] p = 0.02) and WGL (90 min, IQR 40, [40−225] vs 100 min, IQR 30, [55−210], p = 0.02) techniques. Postoperative complication rates were low overall. The highest complication rate among resident-performed surgeries was observed in the CL group, though this difference did not reach statistical significance (24.1% vs 16.2%, p = 0.26). The lowest complication rates for residents were seen in MSL and WGL groups, again without statistical significance respect to attending surgeon (respectively 2% vs 4.6%, p = 0.72 and 2% vs 2.1%, p = 1.00).

**Table 5:** Evaluation of secondary outcomes according to localization technique and operating surgeon.

	ROLL			MSL			WGL			CL		
	Attending surgeon	Resident	p	Attending surgeon	Resident	p	Attending surgeon	Resident	p	Attending surgeon	Resident	p
**CRR**	2.8 (5) [1-78]∗	2 (3) [1-47]∗	0.006	2.7 (3) [1-91]∗	2.3 (4) [[1], [2], [3], [4], [5], [6], [7], [8], [9], [10], [11], [12], [13], [14], [15], [16], [17], [18], [19], [20], [21], [22], [23], [24], [25]]∗	0.77	2.3 (3) [1-32]∗	3.2 (4) [[1], [2], [3], [4], [5], [6], [7], [8], [9], [10], [11], [12], [13], [14], [15], [16]]∗	0.02	3 (4) [1-50]∗	3.7 (2) [[1], [2], [3], [4], [5], [6], [7], [8], [9], [10]]∗	0.41
**Surgery time (min)**												
BCS only	52 (25) [14−200]∗	48 (32) [15-96]∗	0.20	49 (30) [10−208]∗	41 (18) [25-75]∗	0.31	67 (34) [35−180]∗	70 (30) [45−135]∗	0.72	55 (30) [20−250]∗	60 (22) [40−120]∗	0.89
BCS with SLNB	60 (25) [25−189]∗	60 (20) [30−110]∗	0.47	55 (20) [18−180]∗	60 (31) [37-98]∗	0.02	90 (40) [40−225]∗	100 (30) [55−210]∗	0.02	75 (40) [19−180]∗	75 (25) [25−150]∗	0.90
BCS with ALND	100 (40) [50−212]∗	75 (0) [75-75]∗	0.35	66 (57) [30−165]∗	95 (26) [75−110]∗	0.40	120 (55) [95−210]∗	163.5 (41) [143−184]∗	0.43	95 (60) [30−180]∗	N/A	N/A
**Complications**												
**No**	765 (96.2)	142 (94)	0.21	472 (95.4)	50 (98)	0.72	835 (97.9)	144 (98)	1.00	461 (83.8)	22 (75.9)	0.26
**Yes**	30 (3.8)	9 (6)		23 (4.6)	1 (2)		18 (2.1)	3 (2)		89 (16.2)	7 (24.1)	

Values are n (%) unless otherwise indicated; values ∗ are median (i.q.r.) [range]. ROLL = Radioguided Occult Lesion Localization; MSL = Magnetic Seed; WGL= Wire Gide Localization; CL= Carbon Localization; BCS= Breast-conserving surgery; SLNB = sentinel lymph-node biopsy; ALND = axillary lymph-nodes dissection.

## 4 Discussion

This multicenter analysis investigated the impact of surgeon experience on surgical margin status in breast-conserving surgery (BCS) for non-palpable breast lesions, across four widely used localization techniques. Our results indicate that, in a supervised clinical setting, the surgeon's level of experience was not associated with higher margin positivity. This supports the notion that residents, when properly trained and monitored, can safely perform BCS with oncologic outcomes comparable to those of attending surgeons [3,29,30].

The lack of statistically significant differences in margin positivity between residents and attendings reinforces the value of standardized surgical training and real-time supervision. Although a trend toward higher positive margins in resident-performed MSL procedures was observed, this did not reach statistical significance. The complete absence of margin involvement in CL procedures performed by residents—though based on a small sample—further illustrates the variability that can arise from limited case numbers and supports the need for larger cohorts to draw definitive conclusions for each localization technique.

Interestingly, residents achieved significantly better CRR values in ROLL procedures compared to attendings. The reproducibility and reliability of radioguided localization may act as protective factors, effectively levelling the playing field for less experienced surgeons.

We also performed a sub-analysis to assess the rate of infiltrated margins between attending surgeons and residents across different lesion types (microcalcifications, nodules and distortions), with no statistically significant differences observed. These findings indicate that the different morphological characteristics of non-palpable lesions do not influence the performance of either residents or attending surgeons.

Surgical time, as expected, tended to be longer in resident-led procedures. This finding is consistent with prior studies and reflects both the learning phase of technical steps and the deliberate pacing encouraged in teaching settings. However, longer operative times did not correlate with increased complication rates, suggesting that surgical safety is not compromised by extended durations when adequate supervision is provided.

Complication rates were generally low and did not differ significantly between groups. The numerically higher rate observed in resident-performed CL procedures should be interpreted with caution due to the limited number of cases. Notably, complication rates in MSL and WGL cases were equally low across experience levels, further supporting the safety of entrusting residents with these procedures in properly controlled environments.

Taken together, these findings underscore the feasibility and safety of involving surgical trainees in BCS for non-palpable lesions. Localization methods that offer high spatial accuracy and reproducibility, such as ROLL and MSL, may serve as valuable tools for bridging the experience gap.

These findings are aligned with previous studies suggesting that surgical outcomes in teaching hospitals are not compromised when residents operate under close supervision [25,30]. This study has several limitations. First, its retrospective multicenter design introduces the possibility of selection and information bias. Residents performed only a minority of cases, which may have been selectively assigned according to case complexity, resulting in imbalance between groups. In addition, the small number of resident-performed CL cases limited the statistical power for that subgroup.

## 5 Conclusions

This multicenter retrospective analysis demonstrates that, within a supervised clinical setting, the experience level of the operating surgeon—resident versus attending—does not significantly impact surgical margin status, complication rates, or overall surgical performance in breast-conserving surgery for non-palpable breast lesions. While minor variations were observed across different localization techniques, these differences did not translate into inferior oncologic or safety outcomes; in fact, residents achieved a significantly better CRR in ROLL procedures, indicating more conservative resections with maintained surgical quality.

Our findings support the inclusion of surgical trainees in the management of non-palpable breast lesions, particularly when guided by standardized protocols and real-time supervision. Localization techniques with high reproducibility, such as ROLL and MSL, may mitigate the influence of surgical inexperience, ensuring safe and effective treatment even in early stages of training.

These results contribute to the growing body of evidence affirming that surgical education and patient safety can successfully coexist and highlight the importance of maintaining structured training environments.

## Declaration of competing interest

The authors declare that they have no known competing financial interests or personal relationships that could have appeared to influence the work reported in this paper.

## CRediT authorship contribution statement

**Fabio Corsi:** Writing – review & editing, Conceptualization. **Maria Luisa Gasparri:** Data curation. **Sara Albasini:** Writing – original draft, Methodology, Formal analysis, Data curation. **Matilde Pelizzola:** Writing – original draft, Data curation. **Carlo Morasso:** Writing – review & editing, Funding acquisition. **Giulia Armatura:** Data curation. **Alessandro Asaro:** Data curation. **Virginia Casati:** Data curation. **Corrado Chiappa:** Data curation. **Virginia Coli:** Data curation. **Francesca Combi:** Data curation. **Andrea Cuccaro:** Data curation. **Angelica Della Valle:** Data curation. **Raimondo Di Giacomo:** Data curation. **Secondo Folli:** Data curation. **Massimo Maria Grassi:** Data curation. **Stefano Mancini:** Data curation. **Ilaria Maugeri:** Data curation. **Andrea Papadia:** Data curation. **Laura Roveda:** Data curation. **Francesca Rovera:** Data curation. **Silvia Segattini:** Data curation. **Adele Sgarella:** Data curation. **Claudio Siani:** Data curation. **Norma Stefenelli:** Data curation. **Francesco Valenti:** Data curation. **Simone Zanotti:** Writing – review & editing, Data curation, Conceptualization.

## Financial support

This research was partially funded by the European Innovation Council (EIC) through the Horizon Europe programme, under the Grant Agreement No. 101187508 (Spectra BREAST) and by the Italian Ministry of Health (Ricerca corrente program).

## Ethical approval

Data were collected in accordance with a study protocol approved by the Ethical Committee of the coordinating institution (Istituti Clinici Scientifici Maugeri, Pavia, Italy) and of the participating centres.

## Data statement

the data set generated and analysed during the present study is available from the corresponding author upon reasonable request.
